# Cerebral White Matter and Retinal Arterial Health in Hypertension and Type 2 Diabetes Mellitus

**DOI:** 10.1155/2013/329602

**Published:** 2013-07-29

**Authors:** P. L. Yau, R. Hempel, A. Tirsi, A. Convit

**Affiliations:** ^1^Brain, Obesity, and Diabetes Laboratory (BODyLab), Department of Psychiatry, New York University School of Medicine, 145 East 32nd Street, 8th Floor, New York, NY 10016, USA; ^2^Department of Medicine, New York University School of Medicine, 145 East 32nd Street, 8th Floor, New York, NY 10016, USA; ^3^Nathan Kline Institute for Psychiatric Research, 140 Old Orangeburg Road Orangeburg, NY 10962, USA

## Abstract

We examined 33 hypertensive (22 with comorbid type 2 diabetes mellitus (T2DM)) and 29 normotensive (8 with T2DM) middle-aged and elderly adults, comparable in age and education. Relative to normotensive participants, those with hypertension, in addition to a higher prevalence of periventricular white matter (WM) lesions, had significantly lower WM microstructural integrity of major fiber tracts as seen with MRI-based diffusion tensor imaging. Among participants with hypertension, those with co-morbid T2DM (*n* = 22) had more widespread WM pathology than those without T2DM (*n* = 11). Furthermore and consistent with previous research, both hypertension and T2DM were related to decreased retinal arterial diameter. Further exploratory analysis demonstrated that the observed retinal arteriolar narrowing among individual with hypertension was associated with widespread subclinical losses in WM microstructural integrity and these associations were present predominantly in the frontal lobe. We found that T2DM adds to the damaging effects of hypertension on cerebral WM, and notably these effects were independent of age and body mass index. Given that the decrease in retinal arteriolar diameter may be a biomarker for parallel pathology in cerebral arterioles, our data suggest that the frontal lobe may be particularly vulnerable to microvascular damage in the presence of hypertension and T2DM.

## 1. Introduction

Hypertension (HTN) and type 2 diabetes mellitus (T2DM) are common diseases associated with obesity and the metabolic syndrome. Approximately 26 million people in the United States have diabetes and it is estimated that 67% of those individuals also have HTN [1]. Independently of T2DM, HTN affects approximately 30% of the US adult population [2]. HTN and T2DM share several common vascular complications such as coronary artery disease, renal disease, and stroke [3].

HTN and T2DM are strong predictors of cardiovascular disease and can also have a significant impact on brain health [4]. White matter (WM) health is inversely associated with age [5]. Whereas T2DM is frequently associated with volume changes, particularly in the hippocampus [6, 7], both HTN and T2DM are known to result in similarly prominent effects on the integrity of WM [8, 9]. In experimental rodent models, diabetes and HTN have been individually shown to have a differential impact on both cerebral gray and white matter [10]. The impact of HTN on the brain might be further complicated by co-morbid T2DM [6, 11]. 

Given that retinal and cerebral microvessels share developmental and physiological controls [12], it is no surprise that previous studies link retinal vessel integrity to certain aspects of brain health. In particular, narrowed retinal arteriolar diameters are robust indicator of cerebrovascular health [13] and have been found to be a risk factor for HTN [14]. Retinopathy has also been associated with silent brain infarcts among hypertensive individuals [15]. Moreover, there is evidence that retinal vessel diameter is affected by insulin resistance and is related to brain atrophy [16, 17]. However, the role of arterial vessel health in explaining WM integrity in the presence of HTN and/or T2DM remains to be further developed. 

This study had several goals. First, by using a novel and statistically conservative voxelwise analysis method, and after accounting for WM hyperintensities, we wanted to ascertain by using diffusion tensor imaging (DTI) which areas of WM had reductions in microstructural integrity associated with HTN. Secondly, we wanted to evaluate whether T2DM has additive deleterious effects on WM health in the presence of HTN. Finally, given that retinal vessel integrity can be used as a noninvasive marker of cerebral vascular health [17, 18], we also wanted to ascertain whether there are relationships between retinal arteriolar integrity and the anticipated reductions in DTI-based cerebral WM integrity among hypertensive individuals. 

## 2. Methods

### 2.1. Participants

Sixty two middle-aged and elderly community-residing individuals, 33 fulfilling criteria for HTN (see below for definition) and 29 not, were consecutively recruited via web advertisements and referrals from collaborating endocrinologist as part of an ongoing NIH-sponsored study of the effects of diabetes and aging on the brain. All participants had a minimum of a high school education (12–21 years for both groups) and no impairments in their day-to-day functioning and were medically stable (other than some of them having diabetes, hypertension, or dyslipidemia) and free of psychiatric illness or significant vascular disease. Individuals were excluded if they had a history of current insulin treatment, so as to avoid the possible confounding effects of severe hypoglycemic episodes, or if they had uncontrolled hypertension (blood pressure (BP) > 150/90 mm/Hg), significant coronary ischemic disease detected on the electrocardiogram or a Modified Hachinski Ischemia Scale [19] score > 4, any focal neurological signs, current diagnosis or past history of stroke or significant head trauma, or evidence of tumor on the structural MR scan.

In an extensive 8-hour evaluation, performed over 3 separate days, we collected medical, psychiatric, and endocrine data and performed an MRI of the brain. The psychiatric evaluation ensured that our population did not include significant psychiatric illness such as depression or evidence of a degenerative process. The patients were also free of significant vascular disease (Hachinski score below 4; [20]). The New York University School of Medicine Internal Review Board approved this study and each patient gave informed written consent. 

### 2.2. Definition of Diabetes

Individuals with T2DM met one or more of the following criteria: (1) fasting blood glucose ≥6.99 mmol/L on two separate occasions or (2) 2-hour blood glucose level >11.10 mmol/L during a 75 g oral glucose tolerance test or (3) a prior diagnosis of T2DM. Nondiabetic participants had normal fasting glucose levels and showed no evidence of obvious insulin resistance (IR) as demonstrated by their fasting glucose and insulin levels and on the intravenous glucose tolerance test.

### 2.3. Definition of Hypertension

Participants were classified as having HTN if they were receiving anti-HTN pharmacological treatment or had a sitting BP above the NCEP cut-off (systolic BP ≥ 130 mmHg or a diastolic BP ≥ 85 mmHg). BP was determined using a manual sphygmomanometer by a physician during the second visit after an oral or intravenous glucose tolerance test. 

### 2.4. Retinal Image Acquisition and Measurement

The procedure for retinal vessel image acquisition and measurement is detailed elsewhere [16]. Briefly, the participant's eyes are allowed to acclimate to a dimly lit room for 10 minutes in order to nonpharmacologically dilate the pupil sufficiently to view the optic disk and the retinal vessels. Images were focused on the optic disk by having the participant follow a target. A digital image was taken of each eye. The images were then loaded on to a computer, cropped, red-free filtered, and contrast-enhanced by grayscale conversion. 

Using in-house image analysis software an outline of the optic disk was created and the diameter computed. The operator then creates two more concentric circles at 0.5 and 1.0 disc diameters out from the original optic disk outline. This creates two zones, one between the original disc outline and the 0.5 outline and one between the 0.5 outline and the 1.0 outline. The larger of the two zones (between the 0.5 and 1.0 disc outlines) is where the arterioles and venules are identified for measurement. In this zone straight segments of vessel are selected and five equidistant lines orthogonal to the vessel are applied. In an automated fashion, as the vessel is traversed orthogonal to its long axis, the pixel intensity is used to calculate the average width of the vessel. This process is repeated on the 6 largest arterioles and 6 largest venules in the selected zone. To account for branching coefficient, these measurements are combined using the revised Parr-Hubbard formula [21] to produce the central vessel equivalent. In our analyses of these data, we accounted for individual variations in vessel size by residualizing the arteriolar diameters to venular diameters. 

### 2.5. MRI Images

T1-weighted magnetization-prepared rapid acquisition gradient echo (MPRAGE) sequence (TR 1300 ms; TE 4.38 ms; TI 800 ms; FOV 250 × 250; 192 coronal slices; slice thickness 1.2 mm; NEX 1; Flip angle 15°) was used for structural imaging. T2-weighted sequence (TR 9000 ms; TE 94 ms; TI 2000 ms; FOV 210 × 210; 50 axial slices; slice thickness 3 mm) was used to spatially correct DTI images (Please see Section 2.6). In order to account for primary neurological and WM disease, fluid attenuated inversion recovery (FLAIR; TR 9000 ms; TE 97 ms; FOV 210 × 210; 1 average and 2 concatenations; 50 axial slices; slice thickness 3 mm; flip angle 145°) sequence was combined with the MPRAGE and assessed using the modified Fazekas scale [20]. A DTI echo planar sequence (TR 6100 ms; TE 75 ms; delay in TR = 0; b values 0, 1000; 6 directions; FOV 210 × 210; 4 averages and 1 concatenation; 50 axial slices; voxel size 1.64 × 1.64 × 3 mm^3^) was acquired in order to analyze fractional anisotropy (FA).

### 2.6. DTI Voxelwise Image Processing

DTI images were utilized to generate FA maps that were then registered to a structurally relevant MPRAGE image using Automated Registration Toolkit 2 (ART2) as previously described [9, 22]. In short, each structural image was first skull-stripped in native space. These images were then normalized to the Talairach space to obtain a 3D warp field that includes transformation parameters to change the MPRAGE to target image. An MPRAGE transformation matrix was obtained by applying a rigid body registration of the T2-weighted volume to the MPRAGE volume. Another transformation matrix was created to correct for registration errors caused by the participant's head movements. Next, using the skull-stripped T2 image as a guide, the b0 image was iteratively warped to correct for spatial distortions and that produced a 2D warp field with spatial transformation and distortion correction parameters. The FA values and overlay maps were generated from the native DTI images and then these maps were spatially corrected and normalized to standard space using the combination of the four transformations obtained in the previous steps. Furthermore, the FLAIR image, which is very sensitive to WM hyperintensities, was also used as a covariate to control for nonclinically relevant very subtle WM hyperintensities after the image had been normalized to the target template using the same parameters applied to the FA maps.

### 2.7. Statistical Analysis

To examine group differences, independent samples *t*-tests were performed for the demographic and endocrine data. The chi-square test of independence was utilized for data in percentage and nominal variables. In order to adjust for overall vessel diameter, retinal arteriolar diameter was subjected to a linear regression analysis with retinal venular diameter to produce a residual mean of arteriolar diameter that accounts for individual differences in vessel diameter. A two-way factorial ANOVA evaluated the contributions of HTN (HTN versus normotensive) and T2DM (T2DM versus no T2DM) and their interaction effect on retinal arteriolar diameter, after accounting for age and BMI. 

The group FA maps were created by averaging the corrected and normalized FA maps and two-tailed voxelwise analysis of covariance (VANCOVA) analyses were performed with age and WM hyperintensities on the FLAIR image as covariates. Given that HTN is highly associated with obesity [23], we also controlled for BMI in those analyses. The association between WM FA and retinal arteriolar diameter residualized to venular diameter was evaluated among individuals with HTN using a voxelwise correlation analysis with age and BMI as covariates. A WM mask was used to restrict all voxelwise FA analyses to WM regions. In an effort to reduce the likelihood of making type I errors and to adjust for multiple comparisons, we limited the accepted voxels showing statistical significance to those having at least 100 contiguous significant voxels in the same direction (equivalent to at least 0.1 cc in volume). We also chose a conservative false discovery rate of less than one percent utilizing procedures previously described [24]. 

## 3. Results

### 3.1. HTN versus Normotensive

The hypertensive and normotensive groups were well matched on age and did not differ on sex or ethnicity distribution. Descriptors and endocrine data can be found on Table 1. As expected the hypertensive group had significantly higher rates of obesity and T2DM. Despite the fact that 29/33 of our hypertensive participants were on antihypertensive medication, they still had significantly higher systolic but not diastolic BP than the group without HTN. Consistent with the existing literature linking obesity and HTN [23], we found that our hypertensive participants had significantly higher BMI than our normotensive participants. Of those participants who also had a diagnosis of T2DM (22 in the HTN and 8 in the normotensive group, resp.), all but one in the normotensive group were on diabetes medications. The groups did not differ in lipid concentrations, likely due to the fact that significantly more hypertensive participants (22/33) as opposed to only 2/29 in the normotensive group were being treated with cholesterol-lowering medications. 

#### 3.1.1. HTN versus Normotensive: WM FA Comparison

Relative to normotensive participants, those with HTN had significantly higher ratings of periventricular WM hyperintensities (PWMH) and higher, though not statistically significant, ratings of deep WM hyperintensities (DWMH; see Table 1). Additionally, we wanted to explore whether there were subtle changes in WM microstructure even after accounting for those WM hyperintensities seen on the FLAIR image by using a voxelwise DTI FA approach. One control case was excluded from the DTI FA analysis due to uncorrectable spatial distortions on the FA map, leaving 33 hypertensive and 28 normotensive participants for those group comparisons. Since both BP groups had individuals with T2DM, which is associated with an increased incidence of WM lesions, in addition to age, BMI, and the FLAIR image as covariates we also controlled for a diagnosis of T2DM in the DTI FA analyses. 

**Figure 1:**
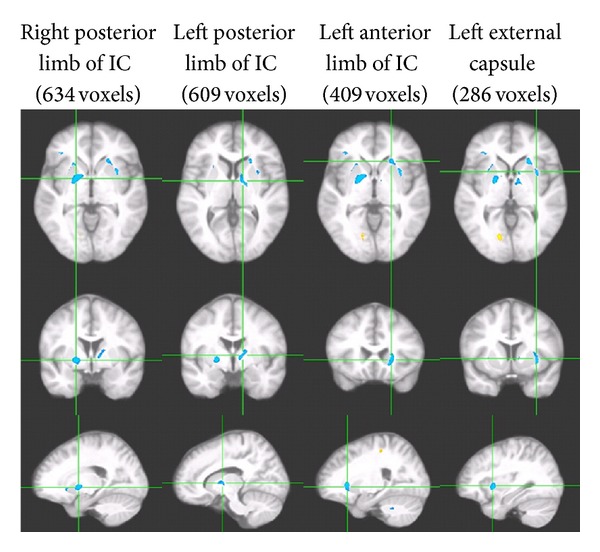
Four largest clusters all demonstrating significant WM FA reductions (clusters in blue) among HTN participants relative to normotensive participants (VANCOVA analyses controlling for age, body mass index (BMI), a diagnosis of T2DM, and the FLAIR image; significance shown for a minimum cluster size of 100 contiguous voxels; *P* < 0.01). Each column shows three orthogonal orientations of the average normalized structural image illustrating a significant cluster with axes passing through the centroid of the cluster (the Talairach space coordinates for the clusters are listed on Table 2).

The result revealed a total of 10 clusters of significant group differences (overall 2,747 voxels or 2.75 cc in volume; *P* < 0.01; Table 2), seven of which showed FA reduction among participants with HTN relative to the normotensive ones (2,344 voxels or 2.34 cc in volume). The largest five clusters showing FA reductions among hypertensive participants were located bilaterally (2,121 voxels) in the internal and external capsules (IC and EC; see Figure 1) and the remaining two were found in the right prefrontal region (114 voxels) and the left cerebellar peduncle (109 voxels). The largest four clusters remained significant at *P* < 0.005. The three clusters showing higher FA values among hypertensive participants were relatively small and were located in regions where there were substantial gray matter contaminations. 

### 3.2. HTN with and without T2DM

To ascertain whether T2DM contributed to further WM damage among hypertensive participants, the hypertensive group was further divided based on whether or not they also had T2DM (22 with HTN and T2DM versus 11 with just HTN; see Table 3 for group descriptors). The subgroups did not differ in age and had similar education and sex distributions. In addition to having significantly higher fasting glucose and hemoglobin A1C (HbA_1C_), the hypertensive group with T2DM also had significantly higher BMI. The groups were comparable in BP, which is not surprising, as individuals with both HTN and T2DM are more likely to receive rigorous pharmacological treatment. All participants with HTN and co-morbid T2DM were receiving antihypertensive medications whereas fewer, 7/11, of those without T2DM were being treated pharmacologically. Despite the fact that more participants with HTN and T2DM were on cholesterol-lowering pharmacological treatment (16/22 versus 6/11), their high-density lipoprotein (HDL) levels were significantly lower (*P* < 0.05) and their triglyceride levels were higher, although not statistically significant, than those of individuals without T2DM (*P* > 0.39).

#### 3.2.1. HTN with and without T2DM-Voxelwise WM FA Group Comparisons

**Figure 2:**
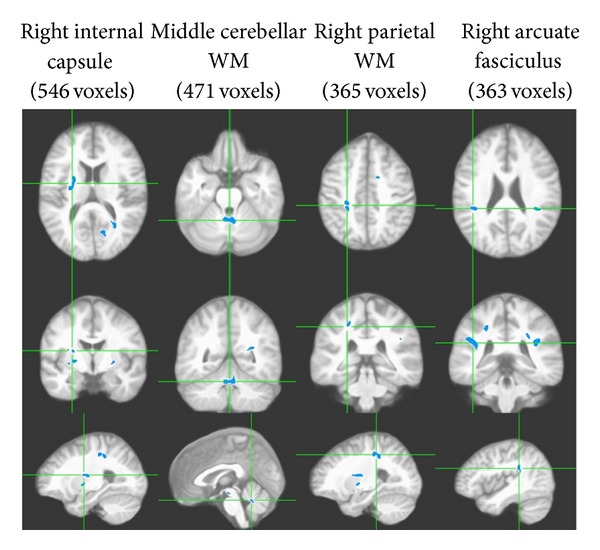
Four (out of 16) clusters all demonstrating significant WM FA reductions among patients with HTN and T2DM compared to those with HTN but no T2DM (VANCOVA analysis controlling for age, BMI, and the FLAIR image; significance shown for a minimum cluster size of 100 contiguous voxels; *P* < 0.01). Each column shows three orthogonal orientations of the average normalized structural image illustrating a significant cluster with the axes passing through the centroid of the cluster (the Talairach space coordinates for the clusters are listed on Table 4).

There were no differences in the Fazekas scores between the groups (see Table 3). Nevertheless, the voxelwise FA comparisons uncovered a total of sixteen clusters all showing WM FA reduction (overall 4,021 voxels or 4.02 cc in volume; *P* < 0.01) among participants with HTN and T2DM relative to those with only HTN, independent of age, BMI, and WM hyperintensities on the FLAIR image, thus suggesting a widespread distribution of WM abnormalities (see Table 4 and Figure 2 for those clusters of ≥200 voxels in size). 

Similar to the results reported above for the HTN yes/no group comparison, significant substantive number of clusters were identified in the IC and EC (total 988 voxels). Also, two clusters were found bilaterally in the arcuate fasciculi (total 645 voxels), which connect the frontal and temporal lobes. Two clusters were identified bilaterally in the parietal lobe (total 686 voxels), two in the left occipital lobe (total 414 voxels), two in the temporal lobe (total 294 voxels), one in the left the corpus callosum (228 voxels), and one in the left frontal lobe (151 voxels). The remaining two clusters were located in the subcortical regions (total 615 voxels).

### 3.3. Associations between WM FA and Retinal Arteriolar Diameter among Participants with HTN

**Figure 3:**
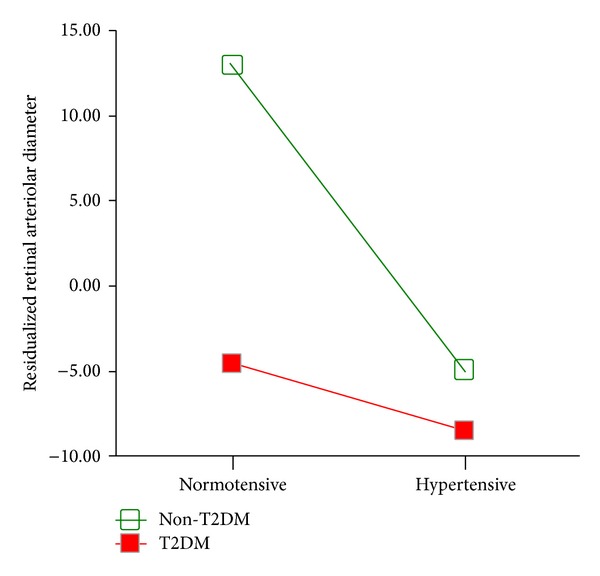
HTN × T2DM interaction effect on residualized retinal arteriolar diameter.

Retinal vessel diameter (arterioles and venules) was measured for all but three participants (two of those excluded had poor retinal image quality and one had cataracts). The factorial ANOVA analysis revealed a significant HTN effect (HTN, 170.98 ± 16.51 *μ*m versus normotensive,184.63 ± 20.43 *μ*m, *F*[1,53] = 7.29, *P* < 0.01, *η*_*p*_^2^ = 0.121), with reduced retinal arteriolar diameter in HTN, showing a medium-large effect size. The T2DM effect was also significant (T2DM, 173.71 ± 18.42 *μ*m versus no T2DM, 180.84 ± 20.23 *μ*m, *F*[1,53] = 5.10, *P* = 0.03, *η*_*p*_^2^ = 0.088), with the effect size being medium-large. Please note that the means and standard deviations in the previous sentence are the raw arteriolar diameters prior to residualization, but the statistics presented were derived from the comparisons of the residualized values. The HTN × T2DM interaction effect was significant as well with a medium-large effect size (*F*[1,53] = 4.13, *P* = 0.05, *η*_*p*_^2^ = 0.072; see Figure 3). Importantly, these effects were all independent of age and BMI. As seen on Figure 3, participants with HTN and/or T2DM had distinctively smaller retinal arteriolar diameter than those without these conditions.

**Figure 4:**
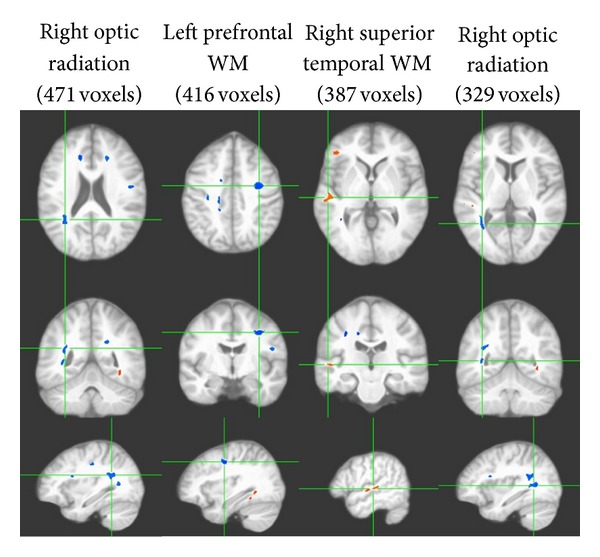
Four largest clusters demonstrating significant associations between FA and residualized arteriolar diameter among patients with HTN (VANCOVA analysis controlling for age and BMI; minimum cluster size of 100 voxels; *P* < 0.01; clusters in deep blue represent positive associations whereas those in orange represent negative associations). Each column shows three orthogonal orientations of the average normalized structural image illustrating a significant cluster with the axes passing through the centroid of the cluster (the Talairach space coordinates for the clusters are listed on Table 5).

In light of these findings, we further explored whether the observed reduction in retinal arteriolar size (residualized to venular diameter) could explain the lower WM microstructural integrity among hypertensive participants. Using the voxelwise correlational approach outlined in the Methods section, we identified a total of 15 clusters showing significant association between the residualized arteriolar diameter and WM FA values (overall 3,748 voxels or 3.75 cc in volume, *P* < 0.01; see Table 5), twelve of which demonstrated significant positive associations (overall 2,846 voxels or 2.85 cc in volume). These correlations were independent of both age and BMI. Five of those clusters remained significant at *P* < 0.005. Nine of the clusters were located in the frontal regions (total 1892 voxels), two in the right optic radiation (total 800 voxels), and one in the left parietal lobe (154 voxels). The four largest clusters demonstrating significant correlation of at least 200 voxels in size are illustrated in Figure 4. The three clusters in the opposite direction, namely, demonstrating significant inverse correlation between FA and residualized arteriolar diameter, were located in right superior temporal WM (387 voxels), left temporal WM (327 voxels), and right prefrontal WM (188 voxels). 

## 4. Discussion

We found that among middle-aged and elderly adults, HTN, in addition to being associated with increased incidence of periventricular WM lesions, also affects the microstructural integrity of major WM fiber tracts. These more diffuse effects are still present despite the fact that 29/33 of the HTN participants were receiving pharmacological antihypertensive treatment and had BP under reasonable control at the time of the study. Moreover, individuals with HTN and co-morbid T2DM manifest larger WM microstructural abnormalities than those with HTN alone. We also demonstrate, for the first time, that the retinal arterial narrowing in HTN is related to extensive reductions in WM microstructural integrity, with the frontal lobe areas being predominantly affected. This is shown in Figure 3, which represents the HTN × T2DM interaction effect on retinal arteriolar width (a measure of arteriolar health). 

We demonstrated the impact of HTN on WM integrity using different measures of WM health. WM hyperintensity assessments using the Fazekas scale showed significantly higher ratings among individuals with HTN, and this is consistent with previous work [25]. We also found significant alterations in cerebral WM microstructural integrity as measured by FA in participants with HTN, and these findings were independent of age, obesity, and the WM hyperintensities seen on the FLAIR image. However, the existing literature is inconsistent in the relationship between FA and HTN. Some studies show no significant differences in whole brain mean FA [26], others found differences in FA only in the optic radiation [27], while some found more dramatic differences in FA in the frontal periventricular and parieto-occipital areas [28]. Here, we found that among participants with HTN there were distinct areas of reduced FA primarily in the IC and EC bilaterally. Our findings may be somewhat different from those previously reported in that both our hypertensive and normotensive groups contained individuals with T2DM; T2DM and HTN likely have different effects on the brain [10]. Nevertheless, in order to adjust for these potential additive effects, we also accounted for a diagnosis of T2DM in our DTI voxelwise HTN group FA comparisons. 

We provide preliminary evidence that participants with both HTN and T2DM have more widespread, albeit subtle, deficits in WM microstructural integrity than those with only HTN, despite being in overall good BP control. Furthermore, these effects were also independent of age, overt WM pathology (hyperintensities present on the FLAIR image), and BMI. To our knowledge, this is the first report on the co-morbid impact of HTN and T2DM on WM microstructure. Although this data set is relatively small and our findings and conclusions should be considered preliminary, determining the combined effects of HTN and T2DM may be valuable towards understanding the full influence of the metabolic syndrome on the brain. The preliminary results presented here are supported by rodent models, where both diabetes and HTN were found to contribute to microstructural WM abnormalities, but the contribution of diabetes may have been larger [10]. 

We found that HTN and T2DM are independently related to decreased retinal arteriolar diameter, after adjusting for vessel (venular) diameter. Our result showed that individuals with HTN and/or T2DM have distinctly smaller retinal arteriolar diameter than those with neither conditions, and the group with both conditions was most affected. Importantly, we demonstrated that the observed retinal arteriolar narrowing was related to widespread subclinical WM pathology as reflected by reductions in the MRI DTI-based FA values, and these abnormalities were found predominately in the frontal lobe. Although we also uncovered small regions of negative associations between retinal arteriolar diameter and WM FA, primarily in the temporal lobe, over 75% of the significant voxels showed positive associations. Given that the subgroups were relatively small, future studies should clarify these relationships with larger samples. 

Given the developmental and functional links between retinal arterioles and brain microvasculature [13], the decrease in retinal arteriolar diameter may suggest that parallel pathology exists in cerebral arterioles, which by disrupting blood flow to the cerebral WM [29] may be partially accounting for the reductions in FA reported here. The added WM pathology from co-morbid T2DM was not surprising given the likely vascular origin for this WM pathology and the emerging evidence of cerebrovascular reactivity deficits, carotid stiffening, and intima-media thickening associated with T2DM and IR [30, 31]. We propose that in T2DM, IR, and associated inflammation contribute to impairments in cerebral vascular reactivity which, when coupled with other adverse mechanisms such as increased oxidative stress, in turn may lead to neuronal and WM damage.

A significant strength of this report is derived from the use of a voxelwise DTI analysis procedure, which allows for precise determination of the location of WM changes utilizing conservative statistics. Previous reports analyzed the DTI based on average FA values of larger structures, which may have overestimated the actual brain abnormalities. A second strength of the study is our very conservative statistical approach; by covarying out age and BMI effects on WM, as well as the overt WM damage seen on the FLAIR image, we were able to capture additional more subtle WM microstructural damage. 

A limitation of this study is the relatively small number of participants, especially in the HTN/T2DM versus HTN only analysis. Nevertheless, despite the relatively small numbers of subjects, the DTI results, which used very conservative statistical thresholds, were consistently in the predicted direction; those subjects with HTN and T2DM had significant reductions in their WM microstructural integrity relative to those with only HTN. Although the relationship between hypertension and cerebral white matter damage may be influenced by ethnicity as previously reported [32], given our relatively small sample size, it was not feasible to split the analysis by ethnicity. However our groups were well balanced on ethnicity. Another limitation is that we do not have information regarding smoking in our participants, and details regarding hypertension and diabetes medications or antiplatelet use were only available for 35/62 of the participants. Antihypertensive medications, such as diuretics, have effects on cerebral autoregulation [33], but only two of our subjects reported diuretic use, so we do not think that this significantly contributed to our findings. Future studies should utilize autoregulation or cerebral blood flow assessments to ascertain their possible effects on WM abnormalities.

In summary, this study suggests that T2DM adds to the damaging effects of HTN on WM microstructural integrity, with the frontal lobes being particularly vulnerable. Ascertaining DTI-based FA values, we demonstrated subclinical diffuse alterations in WM microstructural integrity above and beyond the WM areas showing ischemic changes (hyperintensities). Although we accounted for age in our statistics, this statistical adjustment may not account for differences in subtle age-associated rates of atherosclerosis. Studying these much younger individuals would allow us to differentiate more acute effects from more chronic ones associated with atherosclerosis and/or more advanced forms of vascular disease.

## Figures and Tables

**Table 1:** Demographic and endocrine data: hypertensive versus normotensive.

	Hypertensive	Normotensive	*P*	Cohen's *d*
	(*n* = 33)	(*n* = 29)		
	Mean ± SD	Mean ± SD		
Age (years)	59.70 ± 7.84	56.26 ± 6.41	0.07	0.48
Gender^a^	14 F/19 M	15 F/14 M	0.46	
Ethnicity^a^ (white/black/hispanic/others)	22/7/2/2	20/6/1/2	0.97	
Diabetes (%)^a^	66.7%	27.6%	<0.01	
BMI (kg/m^2^)	30.42 ± 7.28	25.70 ± 4.97	<0.01	0.75
Systolic BP (mmHg)	121.97 ± 14.55	113.83 ± 10.09	0.01	0.64
Diastolic BP (mmHg)	72.94 ± 11.09	71.00 ± 6.57	0.40	0.21
HbA_1C_ (%)	7.24 ± 1.90	6.09 ± 1.54	0.01	0.66
Fasting glucose (mmol/L)	6.81 ± 3.17	5.05 ± 1.30	0.01	0.71
HDL (mmol/L)	2.72 ± 0.92	2.69 ± 0.61	0.91	0.03
Triglycerides (mmol/L)	7.38 ± 5.52	5.66 ± 2.46	0.13	0.40
PWMH rating^a,b^	15/13/5	23/6/0	0.01	
DWMH rating^a,b^	15/13/5	20/8/1	0.12	

Data is provided in means ± standard deviations unless otherwise specified.

^a^Chi-square test of independence for nominal data and data in percentage.

^b^Data represent number of individuals for WMH ratings of 0, 1, and 2, respectively.

**Table 2:** Significant clusters demonstrating FA reduction among HTN relative to normotensive participants (>200 voxels in size; *P* < 0.01).

Clusters	Size (no. of voxels)	*Talairach coordinates *		
		*X*	*Y*	*Z*
Right posterior limb of IC*	634	−18.5	14.8	−20.7
Left posterior limb of IC*	609	13.1	17.8	−13.8
Left anterior limb of IC*	409	22.4	−6.9	−19.6
Left external capsule*	286	32.6	6.3	−18.3

*Remained significant at *P* < 0.005.

**Table 3:** Demographic and endocrine data: hypertensive/T2DM versus hypertensive/non-T2DM.

	HTN/T2DM	HTN/non-T2DM	*P*	Cohen's *d*
	(*n* = 22)	(*n* = 11)		
	Mean ± SD	Mean ± SD		
Age (years)	58.36 ± 7.91	62.38 ± 7.32	0.17	−0.52
Gender^a^	11 F/11 M	3 F/8 M	0.22	
Ethnicity (white/black/hispanic/others)	14/5/1/2	8/2/1/0	0.70	
BMI (kg/m^2^)	32.89 ± 7.03	25.48 ± 5.08	<0.01	1.15
Systolic BP (mmHg)	121.36 ± 11.65	123.18 ± 19.75	0.78	−0.12
Diastolic BP (mmHg)	72.59 ± 8.78	73.64 ± 15.20	0.80	−0.09
HbA_1C_ (%)	7.89 ± 1.90	5.64 ± 0.19	<0.001	1.39
Fasting glucose (mg/dL)	8.01 ± 3.26	4.40 ± 0.62	<0.001	1.33
HDL (mg/dL)	2.50 ± 0.80	3.16 ± 1.03	0.05	−0.75
Triglycerides (mg/dL)	7.97 ± 6.40	6.21 ± 3.04	0.39	0.32
PWMH rating^a,b^	9 /10/3	6/3/2	0.60	
DWMH rating^a,b^	10/8/4	5/5/1	0.76	

Data is provided in means ± standard deviations unless otherwise specified.

^a^Chi-square test of independence for nominal data and data in percentage.

^b^Data represent number of individuals for WMH ratings of 0, 1, and 2, respectively.

**Table 4:** Significant clusters of FA reduction among HTN with T2DM relative to HTN only (>200 voxels in size; *P* < 0.01).

Clusters	Size (no. of voxels)	*Talairach coordinates *		
		*X*	*Y*	*Z*
Right internal capsule*	546	−26.3	21.2	−4.2
Medial cerebellar WM*	471	1.3	68.3	−42.8
Right parietal WM*	365	−24.7	49.0	27.3
Right arcuate fasciculus*	363	−41.9	53.0	6.1
Left parietal WM*	321	27.7	56.7	13.9
Left calcarine fissure WM*	291	12.0	85.7	−1.8
Left arcuate fasciculus*	282	38.6	53.2	7.4
Left corpus callosum*	228	28.0	72.0	−1.9

*Remained significant at *P* < 0.005.

**Table 5:** Significant clusters of association between residualized retinal arteriolar diameter and WM FA reduction in HTN (>200 voxels in size; *P* < 0.01).

Clusters	Size (no. of voxels)	*Talairach coordinates *		
		*X*	*Y*	*Z*
Right optic radiation^∗†^	471	−33.1	64.7	3.7
Left prefrontal WM^∗†^	416	33.5	21.7	24.3
Right superior temporal WM*	387	−54.0	37.1	−16.7
Right optic radiation^∗†^	329	−35.4	70.0	−11.9
Left occipitotemporal WM*	327	36.6	62.5	−28.7
Right frontal WM^†^	317	−16.3	45.7	21.3
Left frontal WM^∗†^	260	45.9	26.7	8.4
Right frontal WM^∗†^	236	−14.0	−13.2	5.4

*Remained significant at *P* < 0.005.

^†^Clusters demonstrating positive associations.
